# Correlation between in hospital stay and EuroSCORE index

**DOI:** 10.1186/1749-8090-8-S1-O164

**Published:** 2013-09-11

**Authors:** R Almeida, M Centenaro

**Affiliations:** 1 Cardiovascular Surgery, Faculdade Assis Gurgacz, Cascavel, Brazil

## Background

Time spent in the intensive care unit (ICU) and hospital are increasingly important when planning care for high complex diseases. The objective is to assess the feasibility of Euroscore as a predictor of length of stay.

## Methods

In a retrospective cross-sectional study, October 2009/11, 65 patients were evaluated after CABG. Demographic data, time of CPB, length in ICU and in hospital, were obtained. Patients were stratified into three groups, according to the EuroSCORE additive index: 0-2 (low risk), 3-5 (intermediate risk) and greater than 5 (high risk).

## Results

Fifty two patients (80%) were male; mean age was 62.20 ± 9.51 years; mean body mass index was 28.01 ± 4.16. The additive EuroSCORE was 3.98 ± 3.00. The low-risk group represented 33.84%, 38.46% the intermediate and 27.69% the high risk. Average length of ICU stay was 3.03 ± 4.38 days and hospital 9.64 ± 6.61 days. Analyzing the group with long stay, 63.63% were female (OR 2.40 95% CI 0.58 to 9.81; x2 0.71, p: 0.39), 81.81% were older than 60 years ( OR 0.34 95% CI 0.12 to 2.15; x2 0.34, p: 0.55), 54.55% had a BMI> 25 kg/m2 (OR 0.40 95% CI 0.10 to 1 , 53; x2 0.99, p: 0.31), 81.81% remained for more than 40 minutes on CPB (OR 0.09 95% CI 0.00 to 1.14; x2 2.11, p: 0 14). In relation to risk stratification, had long stay: the low-risk patients 9.09% (OR 0.35 95% CI 0.06 to 1.81; x2 0.87, p: 0.35), the risk intermediate 12.00% (OR 0.51 95% CI 0.12 to 2.15; x2 0.34, p: 0.53) and 33.33% high risk (OR 5.04 95% CI 1.27 -19.88; x2 4.25, p: 0.03).

## Conclusions

The authors found correlation between the length of stay in ICU and hospital, with the Euroscore index, with statistical significance in the high risk group.

